# Supplementary material for: Analysis of PPARγ Signaling Activity in Psoriasis
Source: Int J Mol Sci. 2021 Aug 10;22(16):8603. doi: 10.3390/ijms22168603 (PMC8395241; doi:10.3390/ijms22168603)
Supplement: Supplementary file 1 [file ijms-22-08603-s001.zip › Supplemental materials_Analysis of PPARg signaling activity in psoriasis/Pathway models/Models images and html files/Model of PPARG related pathways in psoriasis (short version)/6484.html]

IRF3


# Protein IRF3

|  |  |
| --- | --- |
| URN | urn:agi-llid:3661 |
| Total Entities | 0 |
| Connectivity | 2133 |
| Name | IRF3 |
| Description | interferon regulatory factor 3 |
| Notes | This gene encodes a member of the interferon regulatory transcription factor (IRF) family. The encoded protein is found in an inactive cytoplasmic form that upon serine/threonine phosphorylation forms a complex with CREBBP. This complex translocates to the nucleus and activates the transcription of interferons alpha and beta, as well as other interferon-induced genes. Alternatively spliced transcript variants encoding multiple isoforms have been observed for this gene. [provided by RefSeq, Nov 2011] |
| Primary Cell Localization | Nucleus |
| Class | Transcription factor |
| ObjectType | Protein |

---

|  |  |
| --- | --- |
| Pathway | Cortisol in Resolving Inflammation |
|  | Antiviral Signaling through Pattern Recognition Receptors |
|  | TLR4 Signaling in Leukocytes |
|  | Toll-like Receptors in Sterile Inflammation |
|  | Macrophage M1 Lineage |
|  | AXL Receptor Inhibits Macrophages and Dendritic Cells Function |
|  | Dendritic Cells Function in Atherosclerosis |
|  | MAVS in Antiviral Innate Immune Response in Myocarditis |
|  | Proteins Involved in Myocarditis |
|  | Dendritic Cells Function in Psoriasis |
|  | Apoptotic Keratinocytes Clearance Recession in Systemic Lupus Erythematosus |
|  | Dendritic Cell Activation in Systemic Lupus Erythematosis |
|  | Neutrophil and Macrophage Function in Systemic Lupus Erythematosus |
|  | Plasmacytoid Dendritic Cell in Diabetes Mellitus Type 1 |
|  | Crohn's Disease |
|  | Dendritic Cell Dysfunction in Crohn's disease |
|  | Dendritic Cell Function in Ulcerative Colitis |
|  | Airway Epithelial Cell Dysfunction |
|  | Toll-like Receptors (TLR) in Periodontitis |
|  | HPV E6 and E7 Induced Disruption of TNFR/NF-kB Signaling in Keratinocytes |
|  | TLR3 Role in Retinal Pigment Epithelial Cell |
|  | Alveolar Macrophages Dysfunction in COPD |
|  | IL15 Expression Targets |
|  | TLR3 Expression Targets |
|  | TLR4 -> NF-kB/IRF Expression Targets |
|  | TLR4 -> IRF Signaling |
|  | TLR3 -> IRF Signaling |
|  | pathway 4 |
|  | New Pathway (2) |
|  | Model of PPARG signaling in psoriasis |
|  | PPARG negative regulators and targets |
|  | Model of PPARG related pathways in psoriasis (short version) |
|  | sars-cov binding |
|  | sars-cov no binding |
|  | dsRNA induces innate immunity and INFs in alveolar cells |
|  | AGER feed forward loop in innate immunity |
|  | Dendritic Cell Activation in COVID19 |
|  | Overview. Diseases of the genitourinary system (chapter 13) |
|  | 1\_Insufficient activation of immune response in the middle ear epithelium cells in otitis media |
|  | 1\_Defects in response to pathogens in the gut promotes inflammation in Crohn's disease |
|  | 2\_Intestine epithelial cells drive immunological responses in ulcerative colitis |
|  | 1\_Antigen-presenting cells promote islet dysfunction and immune system activation in T1D |

---

|  |  |
| --- | --- |
| Group | Genes Associated with Systemic Lupus Erythematosus |
|  | binding partnetrs for SARSs |
|  | other partners for SARS |

---

|  |  |
| --- | --- |
| MedScan ID | 3661 |

---

|  |  |
| --- | --- |
| LocusLink ID | 3661 |
|  | 54131 |
|  | 292892 |

---

|  |  |
| --- | --- |
| Alias | C920001K05Rik |
|  | OTTMUSP00000022257 |
|  | IRF III |
|  | interferon regulatory factor 3 |
|  | IRF-3 |
|  | IIAE7 |
|  | OTTMUSP00000022256 |
|  | IRF IIIs |
|  | C920001K05 |
|  | interferon regulatory factor III |
|  | OTTMUSP00000022258 |
|  | RIKEN cDNA C920001K05 gene |
|  | MGC94729 |
|  | MGC91046 |

---

|  |  |
| --- | --- |
| GO ID | 0003677 |
|  | 0001228 |
|  | 0000981 |
|  | 0001227 |
|  | 0000978 |
|  | 0019904 |
|  | 0042803 |
|  | 0043565 |
|  | 0003712 |
|  | 0039530 |
|  | 0035666 |
|  | 0006915 |
|  | 0006974 |
|  | 0071360 |
|  | 0051607 |
|  | 0002376 |
|  | 0060333 |
|  | 0031663 |
|  | 0071888 |
|  | 0000122 |
|  | 0032480 |
|  | 0043123 |
|  | 0050715 |
|  | 0032727 |
|  | 0032728 |
|  | 0045944 |
|  | 0032481 |
|  | 0060340 |
|  | 0097300 |
|  | 0050727 |
|  | 0032479 |
|  | 0006366 |
|  | 0045351 |
|  | 0060337 |
|  | 0016032 |
|  | 0005737 |
|  | 0005829 |
|  | 0005654 |
|  | 0005634 |
|  | 0003700 |
|  | 0044212 |
|  | 0071222 |
|  | 0045087 |
|  | 0050689 |
|  | 0045358 |
|  | 0045893 |
|  | 0010468 |
|  | 0006355 |
|  | 0009617 |
|  | 0043330 |
|  | 0032496 |
|  | 0006917 |
|  | 0034142 |
|  | 0006351 |
|  | 0000975 |
|  | 0002756 |
|  | 0001077 |
|  | 0005515 |
|  | 0019221 |
|  | 0034138 |
|  | 0008063 |
|  | 0001078 |
|  | 0071359 |
|  | 0010008 |
|  | 0019048 |
|  | 0042802 |
|  | 0002224 |
|  | 0003702 |
|  | 0044419 |
|  | 0009615 |
|  | 0005886 |
|  | 0010843 |
|  | 0010553 |

---

|  |  |
| --- | --- |
| KEGG ID | hsa:3661 |
|  | mmu:54131 |
|  | rno:292892 |

---

|  |  |
| --- | --- |
| Organism | Homo sapiens {Organism urn:agi-taxid:9606} |
|  | Mus musculus {Organism urn:agi-taxid:10090} |
|  | Rattus norvegicus {Organism urn:agi-taxid:10116} |
|  | Homo sapiens |
|  | Mus musculus |
|  | Rattus norvegicus |

---

|  |  |
| --- | --- |
| Mouse chromosome position | 7 |

---

|  |  |
| --- | --- |
| OMIM ID | 603734 |
|  | 616532 |

---

|  |  |
| --- | --- |
| Rat chromosome position | 1q22 |

---

|  |  |
| --- | --- |
| Hugo ID | 6118 |
|  | HGNC:6118 |

---

|  |  |
| --- | --- |
| Human chromosome position | 19q13.33 |
|  | 19q13.3-q13.4 |

---

|  |  |
| --- | --- |
| Swiss-Prot Accession | Q14653 |
|  | E2GIM5 |
|  | E2GIM7 |
|  | E2GIM8 |
|  | E2GIM9 |
|  | M0QYT9 |
|  | E2GIM6 |
|  | Q14653.1 |
|  | P70671 |
|  | Q3U9K6 |
|  | P70671.1 |
|  | Q5XIB0 |
|  | A8K7L2 |
|  | B2RAZ3 |
|  | Q5FBY1 |
|  | Q5FBY2 |
|  | Q5FBY4 |
|  | Q7Z5G6 |
|  | Q71U93 |
|  | Q96GL3 |
|  | O54726 |
|  | Q3UJM9 |

---

|  |  |
| --- | --- |
| GenBank ID | NC\_000019 |
|  | NR\_045568 |
|  | XR\_001753678 |
|  | XR\_002958310 |
|  | XR\_002958311 |
|  | XR\_002958312 |
|  | NM\_001197125 |
|  | NP\_001184054 |
|  | XM\_017026767 |
|  | XP\_016882256 |
|  | XM\_006723198 |
|  | XP\_006723261 |
|  | XM\_017026766 |
|  | XP\_016882255 |
|  | XM\_006723197 |
|  | XP\_006723260 |
|  | NM\_001197128 |
|  | NP\_001184057 |
|  | NM\_001197126 |
|  | NP\_001184055 |
|  | NM\_001197127 |
|  | NP\_001184056 |
|  | NM\_001197123 |
|  | NP\_001184052 |
|  | NM\_001197124 |
|  | NP\_001184053 |
|  | NM\_001571 |
|  | NP\_001562 |
|  | NM\_001197122 |
|  | NP\_001184051 |
|  | XM\_024451497 |
|  | XP\_024307265 |
|  | XM\_024451498 |
|  | XP\_024307266 |
|  | XM\_024451495 |
|  | XP\_024307263 |
|  | XM\_024451494 |
|  | XP\_024307262 |
|  | XM\_006723201 |
|  | XP\_006723264 |
|  | XM\_024451496 |
|  | XP\_024307264 |
|  | XM\_024451493 |
|  | XP\_024307261 |
|  | XM\_024451492 |
|  | XP\_024307260 |
|  | XM\_006723200 |
|  | XP\_006723263 |
|  | NG\_031810 |
|  | AC011495 |
|  | AF112181 |
|  | AAD05262 |
|  | CH471177 |
|  | EAW52510 |
|  | EAW52511 |
|  | EAW52512 |
|  | EAW52513 |
|  | U86636 |
|  | AAC68818 |
|  | AA568913 |
|  | AB102884 |
|  | BAD89413 |
|  | AB102886 |
|  | BAD89415 |
|  | AB102887 |
|  | BAD89416 |
|  | AK057577 |
|  | AK292027 |
|  | BAF84716 |
|  | AK314421 |
|  | BAG37040 |
|  | BC000660 |
|  | AAH00660 |
|  | BC009395 |
|  | AAH09395 |
|  | BC071721 |
|  | AAH71721 |
|  | BM477213 |
|  | BP347627 |
|  | BU859806 |
|  | CB961512 |
|  | DA938709 |
|  | DA999381 |
|  | HM357932 |
|  | ADN86147 |
|  | HM357933 |
|  | ADN86148 |
|  | HM357934 |
|  | ADN86149 |
|  | HM357935 |
|  | ADN86150 |
|  | HM357936 |
|  | ADN86151 |
|  | HM357937 |
|  | ADN86152 |
|  | Z56281 |
|  | CAA91227 |
|  | Q14653 |
|  | NC\_000073 |
|  | NR\_045611 |
|  | NM\_016849 |
|  | NP\_058545 |
|  | XM\_006540995 |
|  | XP\_006541058 |
|  | XM\_006540996 |
|  | XP\_006541059 |
|  | AC155806 |
|  | AF036341 |
|  | AAC68814 |
|  | CH466603 |
|  | EDL22782 |
|  | EDL22783 |
|  | EDL22784 |
|  | EDL22785 |
|  | EDL22786 |
|  | AK013944 |
|  | AK050570 |
|  | AK145977 |
|  | BAE26800 |
|  | AK146378 |
|  | BAE27126 |
|  | AK151750 |
|  | BAE30660 |
|  | AK152689 |
|  | BAE31420 |
|  | BC003233 |
|  | BC023244 |
|  | BC050882 |
|  | AAH50882 |
|  | BY089035 |
|  | EU545481 |
|  | ACD67887 |
|  | U75839 |
|  | AAB36924 |
|  | U75840 |
|  | AAB36925 |
|  | P70671 |
|  | NC\_005100 |
|  | XM\_006229021 |
|  | XP\_006229083 |
|  | XM\_008759337 |
|  | XP\_008757559 |
|  | XM\_006229022 |
|  | XP\_006229084 |
|  | XM\_006229023 |
|  | XP\_006229085 |
|  | NM\_001006969 |
|  | NP\_001006970 |
|  | XM\_006229024 |
|  | XP\_006229086 |
|  | AC\_000069 |
|  | AAHX01004996 |
|  | AC127719 |
|  | CH473979 |
|  | EDM07429 |
|  | EDM07430 |
|  | EDM07431 |
|  | EDM07432 |
|  | BC083776 |
|  | AAH83776 |
|  | FQ213393 |
|  | FQ218848 |
|  | AAHY01061132 |
|  | XM\_006723199 |
|  | ABM84381 |
|  | XR\_935819 |
|  | ABM87305 |
|  | XR\_935820 |
|  | AC\_000029 |
|  | XR\_430199 |
|  | AC\_000151 |
|  | DQ893455 |
|  | XM\_006723202 |
|  | NC\_018930 |
|  | DQ896306 |
|  | AMYH02036325 |
|  | XP\_006723265 |
|  | ABBA01032913 |
|  | AABR06004808 |
|  | XP\_006723262 |
|  | NT\_011109 |
|  | NW\_001838497 |
|  | NW\_927240 |
|  | AC\_000062 |
|  | CR590897 |
|  | CR596241 |
|  | CR608119 |
|  | CR613061 |
|  | CR613940 |
|  | CR615920 |
|  | CR624964 |
|  | Q5FBY1 |
|  | Q5FBY2 |
|  | Q5FBY4 |
|  | Q71U93 |
|  | Q7Z5G6 |
|  | Q96GL3 |
|  | NT\_039424 |
|  | NW\_001030849 |
|  | O54726 |
|  | Q3U9K6 |
|  | Q3UJM9 |
|  | NW\_001084763 |
|  | NW\_047558 |
|  | Q5XIB0 |

---

|  |  |
| --- | --- |
| Swiss-Prot ID | IRF3\_HUMAN |
|  | IRF3\_MOUSE |
|  | E2GIM8\_HUMAN |
|  | Q7Z5G6\_HUMAN |
|  | Q3U9K6\_MOUSE |
|  | E2GIM7\_HUMAN |
|  | E2GIM6\_HUMAN |
|  | Q5XIB0\_RAT |
|  | E2GIM5\_HUMAN |
|  | E2GIM9\_HUMAN |
|  | Q5FBY2\_HUMAN |
|  | Q5FBY1\_HUMAN |

---

|  |  |
| --- | --- |
| Cell Localization | Cytoplasm |
|  | Nucleus |

---

|  |  |
| --- | --- |
| Ensembl ID | ENSG00000126456 |
|  | ENSP00000471896.1 |
|  | ENST00000601291.5 |
|  | ENSP00000471358.1 |
|  | ENST00000599223.5 |
|  | ENSP00000366339.5 |
|  | ENST00000377135.8 |
|  | ENSP00000470069.1 |
|  | ENST00000599144.5 |
|  | ENSP00000472700.1 |
|  | ENST00000600022.5 |
|  | ENSP00000470512.1 |
|  | ENST00000596765.5 |
|  | ENSP00000366344.3 |
|  | ENST00000377139.8 |
|  | ENSP00000310127.6 |
|  | ENST00000309877.11 |
|  | ENSP00000470431.1 |
|  | ENST00000600911.5 |
|  | ENSP00000469113.1 |
|  | ENST00000597198.5 |
|  | ENSP00000472582.1 |
|  | ENST00000598808.5 |
|  | ENSMUSG00000003184 |
|  | ENSMUSP00000003284.8 |
|  | ENSMUST00000003284.15 |
|  | ENSMUSP00000146773.1 |
|  | ENSMUST00000209066.1 |
|  | ENSMUSP00000103465.1 |
|  | ENSMUST00000107834.1 |
|  | ENSRNOG00000043388 |
|  | ENSRNOP00000027786.4 |
|  | ENSRNOT00000027786.6 |
|  | ENST00000596765 |
|  | ENSRNOP00000027786 |
|  | ENSMUSP00000003284 |
|  | ENST00000600022 |
|  | ENSP00000471358 |
|  | ENSP00000472601 |
|  | ENSP00000470069 |
|  | ENSMUST00000107835 |
|  | ENSMUST00000107834 |
|  | ENSP00000366344 |
|  | ENSMUSP00000103466 |
|  | ENSMUSP00000103465 |
|  | ENST00000377135 |
|  | ENST00000597636 |
|  | ENSP00000472582 |
|  | ENST00000377139 |
|  | ENSRNOT00000027786 |
|  | ENSMUST00000003284 |
|  | ENST00000598808 |
|  | ENSP00000472700 |
|  | ENSP00000471896 |
|  | ENSP00000366339 |
|  | ENST00000309877 |
|  | ENST00000377139.7 |
|  | ENST00000601291 |
|  | ENSMUST00000209066 |
|  | ENST00000599144 |
|  | ENSP00000310127 |
|  | ENST00000599223 |
|  | ENSMUSP00000146773 |
|  | ENST00000593922 |
|  | ENSP00000470512 |

---

|  |  |
| --- | --- |
| MGI ID | MGI:1859179 |
|  | 1859179 |

---

|  |  |
| --- | --- |
| RGD ID | 1549774 |

---

|  |  |
| --- | --- |
| Unigene ID | Hs.75254 |
|  | Hs.731922 |
|  | Mm.489648 |
|  | Rn.1499 |
|  | Hs.289052 |
|  | Mm.3960 |

---

|  |  |
| --- | --- |
| Homologene ID | 1208 |

---

|  |  |
| --- | --- |
| Shape | O-vertex |

---

|  |  |
| --- | --- |
| IPI ID | IPI00111836 |
|  | IPI00291901 |
|  | IPI00737251 |
|  | IPI00646709 |
|  | IPI00976695 |
|  | IPI00364320 |
|  | IPI00941119 |
|  | IPI00873861 |

---
